# ^111^In-DANBIRT *In Vivo* Molecular Imaging of Inflammatory Cells in Atherosclerosis

**DOI:** 10.1155/2018/6508724

**Published:** 2018-11-13

**Authors:** Roberto Mota, Matthew J. Campen, Matthew E. Cuellar, William S. Garver, Jacob Hesterman, Mohammed Qutaish, Tamara Daniels, Monique Nysus, Carston R. Wagner, Jeffrey P. Norenberg

**Affiliations:** ^1^Radiopharmaceutical Sciences, University of New Mexico (UNM), Albuquerque, NM, USA; ^2^Department of Surgery, Division of Vascular Surgery, University of North Carolina at Chapel Hill, Chapel Hill, NC, USA; ^3^Pharmaceutical Sciences, UNM, Albuquerque, NM, USA; ^4^Medicinal Chemistry, College of Pharmacy, University of Minnesota, Minneapolis, MN, USA; ^5^Department of Biochemistry & Molecular Biology, School of Medicine, UNM, Albuquerque, NM, USA; ^6^InviCRO, Boston, MA, USA; ^7^Department of Anesthesiology and Critical Care Medicine, School of Medicine, UNM, Albuquerque, NM, USA

## Abstract

Atherosclerosis-related morbidity and mortality remain a global concern. Atherosclerotic disease follows a slow and silent progression, and the transition from early-stage lesions to vulnerable plaques remains difficult to diagnose. Inflammation is a key component of the development of atherosclerotic plaque and consequent life-threatening complications. This study assessed ^111^In-DANBIRT as an *in vivo*, noninvasive SPECT/CT imaging probe targeting an inflammatory marker, Lymphocyte Function Associated Antigen-1 (LFA-1), in atherosclerotic plaques. *Methods*. Selective binding of ^111^In-DANBIRT was assessed using Sprague-Dawley rats exposed to filtered air and ozone (1 ppm) by inhalation for 4 hours to induce a circulating leukocytosis and neutrophilia in peripheral blood. After 24 hours, whole blood was collected and incubated with radiolabeled DANBIRT (^68^Ga-DANBIRT and ^111^In-DANBIRT). Isolated cell component smeared slides using cytospin technique were stained with Wright-Giemsa stain. Apolipoprotein E-deficient (apoE^−/−^) mice were fed either a normal diet or a high-fat diet (HFD) for 8 weeks. Longitudinal SPECT/CT imaging was performed 3 hours after administration at baseline, 4, and 8 weeks of HFD diet, followed by tissue harvesting for biodistribution, serum lipid analysis, and histology. 3D autoradiography was performed in both groups 24 hours after administration of ^111^In-DANBIRT. *Results*. Increased specific uptake of radiolabeled DANBIRT by neutrophils in the ozone-exposed group was evidenced by the acute immune response due to 4-hour ozone exposure. Molecular imaging performed at 3 hours using SPECT/CT imaging evidenced an exponential longitudinal increase in ^111^In-DANBIRT uptake in atherosclerosis lesions in HFD-fed mice compared to normal-diet-fed mice. Such results were consistent with increased immune response to vascular injury in cardiovascular and also immune tissues, correlated by 24 hours after administration of 3D autoradiography. Histologic analysis confirmed atherosclerotic disease progression with an increased vascular lesion area in HFD-fed mice compared to normal-diet-fed mice. *Conclusion*. ^111^In-DANBIRT is a promising molecular imaging probe to assess inflammation in evolving atheroma and atherosclerotic plaque.

## 1. Introduction

Atherosclerosis is a chronic cardiovascular disease common in patients exhibiting hypercholesterolemia and other inflammatory risk factors [1]. Prevention and early diagnosis of atherosclerotic disease is a top priority in modern medicine [2], as early lifestyle and medical interventions can slow the rate of atheroma development, potentially averting adverse cardiovascular sequelae. Early diagnosis remains challenging because symptoms become clinically evident at later stages where prevention or reversal of lesions is no longer possible [3]. Inflammation promotes continued vascular remodeling in late stage atheroma, making plaques unstable and vulnerable to rupture or erosion. Pathological plaque rupture is the leading cause of acute coronary syndromes, identified by thrombogenic processes and immune cell infiltration [4]. Histological changes in vulnerable plaques such as thin fibrous cap, intraplaque hemorrhage ,and/or a lipid-rich necrotic core (LRNC) are present in ∼80% of ruptured plaques, but such pathologic features require prolonged and variable time of progression [5].

Cardiovascular inflammation [6] and remodeling due to atherogenic progression [7] have been identified as leading causes of plaque instability [8]. The apoE^−/−^ mouse model fed a high-fat diet (HFD) exhibits rapid development of atheromatous plaques [9], with features and stages that mirror those of human disease [10]. Early stages of atherosclerosis typically include altered homeostasis and activation of vascular endothelium, typified by loss of nitric oxide generation and increased expression of chemokines and adhesion molecules [11], which is evident in the apoE^−/−^ model [12]. T cells and monocytes are recruited to plaques and perivascular regions, but their intraplaque specific roles have only been studied invasively postresection or postmortem imaging [13–15]. Neutrophils, part of the immediate innate response of the immunity, have been identified to have an important role in atherosclerosis development [16, 17] and plaque vulnerability [18]. Accumulation of immune cells has been identified in the early-stage lesion of atheroma where it has been published that enzymes such as matrix metalloproteinase [19, 20] and cathepsins play a major role in the atherosclerosis inflammatory process [21]. Experimental models of ozone exposure are used to recreate acute inflammation in animal experimental models to increase systemic inflammatory chemokines and cellularity [22–24]. DANBIRT (DOTA-butylamino-NorBIRT) (Supplemental Figure 1) was developed by chemical repurposing of BIRT 377, which is a specific therapeutic agent developed for leukemia and lymphoma, as it targets Lymphocyte Function Associated Antigen-1 (LFA-1) on both B and T cells [25–27]. DANBIRT is a small nonionic allosteric inhibitor of LFA-1 [28], an integrin expressed only in leukocytes [25], critical for initiation of a vascular immune response to injury [29, 30]. LFA-1 interacts mostly with the intracellular adhesion molecule-1 (ICAM-1) on endothelial cells to promote leukocyte arrest and transmigration [30]. The interaction between LFA-1/ICAM-1 is mediated by chemokines, and LFA-1 affinity is highly modulated whenever LFA-1 interacts/binds to ICAM-1 [31, 32]. Our laboratory has radiolabeled DANBIRT with various radioisotopes suitable for *in vivo* SPECT/PET/CT imaging in small animals [33, 34].

The aim of this study is to evaluate *in vivo* ^111^In-DANBIRT molecular imaging of LFA-1 expressing inflammatory leukocytes within the evolving atheroma as a marker of disease progression in the apoE-deficient mice with either a normal diet or a high-fat diet. Further, the amount of uptake of ^111^In-DANBIRT will characterize the degree of inflammation inside the vascular atherosclerotic plaque of apoE-deficient mice fed a HFD, known to demonstrate rapid progression of high-degree atherosclerosis.

## 2. Materials and Methods

### 2.1. Radiolabeling DANBIRT with ^111^In and ^68^Ga

DANBIRT (M.W. 886.5 G/mole) was effectively synthesized and radiolabeled using ^111^InCl_3_ and ^68^GaCl_3_ as described by Poria et al. with a specific activity of 23 Gbq/pM and a radiolabeling concentration of 1 *µ*g:1 *µ*L [33]. ^111^Indium chloride was obtained from GE Healthcare, and ^68^GaCl_3_ was obtained as an eluate from a ^67^Ge/^68^Ga generator (Eckert and Ziegler). Radionuclide incorporation yield and radiochemical purity of radiolabeled DANBIRT were characterized by instantaneous thin layer chromatography (ITLC) and high-performance liquid chromatography (HPLC), respectively, following previously published methods [33].

### 2.2. *In Vitro* Stability of Radiolabeled DANBIRT

Radioligand stability of ^68^Ga-DANBIRT was assessed by incubating radiolabeled DANBIRT in two different solutions, Fetal Bovine Serum (FBS) and 0.9% sodium chloride (NaCl), both at 37°C. The incorporation yield (ITLC) and radiochemical purity (HPLC) were assessed at baseline, 5, 10, 30, 60, 120, and 240 minutes performed in triplicate replicates and calculated individually per sample using our previously published methods [33] and ultra-performance liquid chromatography (UPLC) as described in Supplemental Table 1.

### 2.3. Animal Studies

Whole blood was collected for *in vitro* and *ex vivo* studies from male Sprague-Dawley rats (Taconic) aged 6–8 weeks given standard food and water ad libitum. The atherosclerosis model employed male apoE^−/−^ mice (Taconic) aged 6 weeks fed either a normal diet or a high-fat diet (HFD, Harlan-Teklad #88317) for 8 weeks; food and water were provided ad libitum. Animal experiments are summarized in Supplemental Table 2 and were performed in compliance with the Institutional Animal Care and Use Committee (IACUC).

### 2.4. ^111^In-DANBIRT Biodistribution

Mice received 26 Mbq of ^111^In-DANBIRT intravenously (IV) after 8 weeks of HFD or normal diet (*n* of 4 per group). The following organs were harvested and weighed at necropsy: whole blood, tail, aorta, liver, muscle, and adipose tissue. Whole blood was centrifuged, and serum, red blood cells (RBC), and leukocytes were collected. ^111^In-DANBIRT uptake was determined using a gamma counter. Uptake was decay-corrected, and percent-injected activity per gram (%IA/gr) was determined.

### 2.5. ^68^Ga-DANBIRT Biodistribution in Blood Components

As a positive control for a model of acute systemic inflammation, rats were exposed to either filtered air (FA) or ozone (1.0 ppm) for 4 hours (*n* of 3 per group) using a silent arc discharge ozone generator (OREC, Osmonics) to increase circulating proinflammatory cytokines, acute leukocytosis, and neutrophilia [33]. Whole blood was collected 24 hours after exposure, and 5% hetastarch was added to whole blood prior to running isolation protocols. Histopaque neutrophil isolation protocol (10771; Sigma Aldrich) was performed; a modified protocol was used to isolate peripheral blood mononuclear cells (PBMCs) [24]. Individual isolated samples of serum, isolated RBC, neutrophils, and PBMCs in triplicates were washed 3 times using 1x PBS and resuspended in 1% gelatin prior to 1-hour incubation with ^68^Ga-DANBIRT (23 Gbq/pM). After incubation, sample uptake was quantified using a gamma counter (Wallac Wizard 2; Perkin Elmer). Activity was normalized to number of cells in each sample. Cells from isolated samples were centrifuged for 15 minutes at 1000 g at 4°C for smear preparation. Slides were fixed with 5% methanol and stained using a Wright-Giemsa horizontal staining protocol (SLBN4704V; Sigma Aldrich). Cell morphology, differentials, and sample purity were assessed using a light microscope (40x objective BX51; Olympus).

### 2.6. ^111^In-DANBIRT SPECT/CT Imaging

Small-animal SPECT/CT imaging was performed 3 hours after IV injection via tail vein of 26 Mbq ^111^In-DANBIRT at baseline, and after 4 and 8 weeks of HFD or normal diet on a dedicated small animal imaging system (NanoSPECT/CT®; Bioscan, parameters in Supplemental Table 3) for 45-minute acquisition time under deep anesthesia using isoflurane (Piramidal Healthcare, NDC 66794-093-25). Regions of interests (ROIs) were identified using high-quality CT scan with coregistered SPECT/CT 3D reconstruction to accurately locate the tissues of interest (VivoQuant 2.00 software, Invicro) and validated by a veterinary pathologist. The ROIs were extrapolated from the reconstructed CT image and adjusted according to specific morphologic parameters for each mouse tissue such as the aortic arch, descending aorta, and muscle. Concentration and sum per volume normalized to muscle was determined to eliminate ROI signal interference from adjacent tissues. Activity was decay-corrected and compared between dietary groups (*n* of 4 per group).

### 2.7. ^111^In-DANBIRT 3D Autoradiography Imaging

^111^In-DANBIRT 3D autoradiography was performed 24 hours after IV injection via tail vein of 26 Mbq ^111^In-DANBIRT after 8 weeks on either normal diet or HFD. Autoradiography will enhance SPECT/CT imaging and correlate these findings by allowing functional and advanced anatomical assessment of the presence of LFA-1 expression in cardiovascular tissues. Mice were euthanized by CO_2_, and carcasses were individually frozen and embedded vertically in an aqueous solution of 5% carboxymethylcellulose sodium salt (CMC) in a hexane/dry ice bath. Each hexane-frozen block was stored at −20°C prior to mounting on a specimen stage in a cryomacrotome (CM3600 X; Leica). Holes were drilled in CMC blocks adjacent to the carcasses and filled with black India ink and ^14^C (37 kbq/mL) to provide optical and fiducial registration marks. Digital white light photographs (EOS 70D; Canon) were taken of the block surface, 50 *µ*m thick sections in the vertical plane, from the occipital bone to the diaphragm. After every 10 slices, a section was transferred to 2.5 mm tape and dehydrated in the cryomacrotome at −20°C for 24 hours. Dehydrated sections were mounted on black cardboard with calibration standards, covered with 1.4 *µ*m isotope imaging film (FlushTec), placed on imaging plates (Fujifilm; GE Healthcare) and exposed for 24 hours. Screens were imaged on a phosphorous imager (Typhoon FLA 7000; GE Healthcare). Autoradiography and white light files were compiled and ROIs analyzed (VivoQuant 2.00 software, inviCRO) for thymus, muscle, carotid arteries, and aortic arch, as described previously in the 3-hour ^111^In-DANBIRT SPECT/CT imaging methods.

### 2.8. Serum Lipid Levels and Subparticle Analysis

After the last imaging time point, mice were euthanized and whole blood was collected by direct cardiac puncture using heparinized syringes and centrifuged. Serum was analyzed for cholesterol, triglyceride, and size-dependent subparticle quantification (Liposearch; Skylight Biotech).

### 2.9. Histopathology

Mouse hearts were collected at necropsy, perfused, and snap frozen in liquid nitrogen. The upper third of the heart with aortic outflow tract was mounted in optimal cutting temperature (OCT) compound. 10 µm thick sections of the subaortic leaflet region were collected at −20^o^C (Cryostat CM 3050S; Leica). Sections were mounted on slides, incubated with Oil Red O (Sigma-Aldrich, SLBP5248V), and counterstained with Mayer's hematoxylin (Sigma-Aldrich, SLBPG176V). Surface lesion area and arterial vessel circumference were quantified using cellSens Standard 1.13 (Olympus software) to calculate percentage of atheroma lesion area normalized to arterial wall circumference (*n* of 4 per group).

### 2.10. Immunohistochemical Analysis of Intraplaque Expression of CD11a+ (LFA-1+)

The OCT-embedded sections were fixed in methanol, washed, and incubated with fluorescein isothiocyanate conjugated (FITC) rat anti-mouse CD11a antibody 1 : 250 (SouthernBiotech) for 2 hours in a humidified chamber. Sections were counterstained with DAPI (1:1000) and imaged at 10x and 63x using a fluorescence microscope (Zeiss AxioObserver-Hamamatsu Flash4.0 sCMOS Monochrome Camera). Image processing was performed using cellSens Standard 1.13 (Olympus Software).

### 2.11. Statistical Methods

Data were analyzed using two-tailed Student's *t*-test, one-way ANOVA, and repeated measures ANOVA. Tukey's post hoc test and Sidak's correction test were used for multiple comparisons, and the mean differences in quantitative uptake of 3D autoradiography results were compared as %IA/gr of radiolabeled DANBIRT, normalized to muscle. Data are represented as mean ± standard error (SE) unless otherwise indicated. Resulting *p*-values of <0.05 were considered significant (*n* of 4 per group). Prism 6.0 (GraphPad Software) was used for statistical analyses.

## 3. Results

DANBIRT was efficiently radiolabeled for all experiments using both radioisotopes ^111^In (*in vivo* and *ex vivo* studies) and ^68^Ga (*in vitro* studies), achieving ≥95% mean incorporation yield and radiochemical purity. To illustrate radiolabeled DANBIRT *in vitro* stability, samples were incubated with FBS and 0.9% NaCl, and both showed >95% mean radiochemical purity and incorporation yield throughout 240 minutes (Supplemental Figures 2(a) and 2(b)).

### 3.1. *Ex Vivo* Biodistribution of ^111^In-DANBIRT in Atherosclerosis-Prone Mice

**Figure 1:**
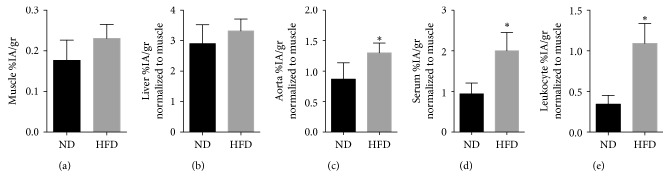
*In vivo* biodistribution of ^111^In-DANBIRT. apoE^−/−^ mice after 8 weeks of dietary assessment were injected with 26 Mbq (∼700 *μ*Ci) of ^111^In-DANBIRT. After 3 hours after injection, mice were euthanized and tissues were harvested for radioactive uptake analysis. Muscle (a) demonstrated low uptake, as expected (determined as the background tissue for other tissues uptake normalization). High uptake in the liver (b) was observed, due to high blood distribution and an abundance of immune cells. Results showed higher uptake in the aorta, serum, and leukocytes (c–e) in mice that were fed a HFD (^*∗*^ *p* < 0.05). Two-tailed Student's *t*-test was used for statistical analysis (^*∗*^ *p* < 0.05) (*n* of 4 per group).

The biodistribution of ^111^In-DANBIRT *ex vivo* in apoE^−/−^ mice receiving a normal diet versus a high-fat diet was evaluated. Results reflected an overall increase in all tissues of ^111^In-DANBIRT in HFD-fed apoE^−/−^ mice compared to normal-diet-fed mice (*p* < 0.05) (Figure 1). Numerous possible mechanisms may explain this finding, such as altered overall excretion, elevated systemic inflammation, or nonspecific sequestration of the tracer into tissues; because the finding was unexpected, the underlying mechanism was not further pursued in this study. Target tissues were normalized to muscle which showed low uptake in both groups (Figure 1(a)). The tissue with the highest uptake was the liver (Figure 1(b)), regardless of dietary supplementation, showing no significant difference between groups. Liver inspection after dissection also revealed a pale white color with fibrous consistency in all HFD-fed apoE^−/−^ mice, absent in normal-diet-fed apoE^−/−^ mice, suggestive of hepatic steatosis. The aorta (Figure 1(c)) reflected higher uptake in apoE^−/−^ mice fed a HFD compared to normal diet (*p* < 0.05). ^111^In-DANBIRT uptake in isolated serum samples (Figure 1(d)) showed higher uptake in HFD-fed mice compared to normal diet while exhibiting higher uptake levels compared to the other blood components. Similar findings were observed in leukocytes with increased uptake in HFD-fed apoE^−/−^ mice (*p* < 0.05) (Figure 1(e)).

### 3.2. Neutrophil Uptake of Radiolabeled DANBIRT in Acute Systemic Inflammatory Cells

**Figure 2:**
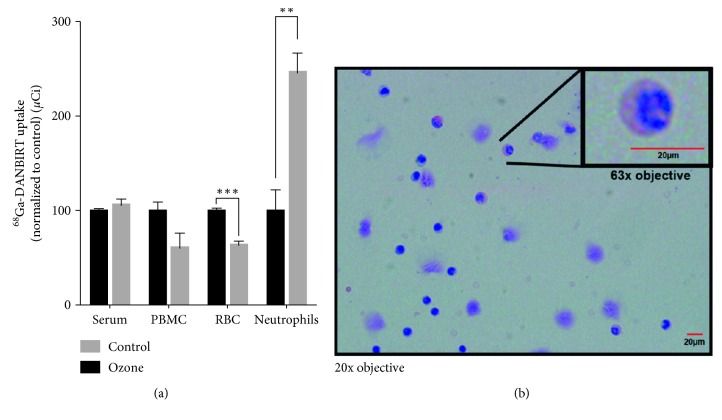
Neutrophil-specific LFA-1 targeting using radiolabeled DANBIRT. Adult rats were exposed to filtered air or ozone for 4 hours, and blood components were isolated afterwards. An acute increase of neutrophils was evidenced uniquely when exposed to ozone (a). Increased uptake was seen in neutrophils and decreased uptake was seen in RBC's after ozone exposure. Wright-Giemsa staining was used in blood smears which particularly evidenced a predominance of immature neutrophils (b) in ozone-exposed rats. One-way ANOVA with multiple comparisons was used for statistical analysis (^*∗∗*^ *p* < 0.01; ^*∗∗∗*^ *p* < 0.001) (*n* of 3 per group).

Biodistribution results showed that mice fed a HFD had increased uptake compared to normal-diet-fed mice (Figure 1). Blood obtained from rats exposed to ozone was used as a positive control to identify leukocytes in whole blood having higher uptake of ^111^In-DANBIRT. ^68^Ga-DANBIRT uptake increased in neutrophils (^*∗*^ *p*=0.008) and unexpectedly decreased in RBC (Figure 2(a)) with no significant differences in serum or PBMC. A predominance of immature neutrophils (∼59%) (Figure 2(b)) and PBMC (∼53%) were observed in ozone-exposed rats, correlating with induced circulating leukocytosis with specific neutrophilia, without other relevant morphologic cellular changes, as previously described [24]. This demonstrates that under acute systemic inflammatory conditions, LFA-1 affinity in neutrophils is increased and therefore a target for *in vivo* molecular imaging.

### 3.3. Characterization of Vascular Atherosclerotic Mouse Model

**Figure 3:**
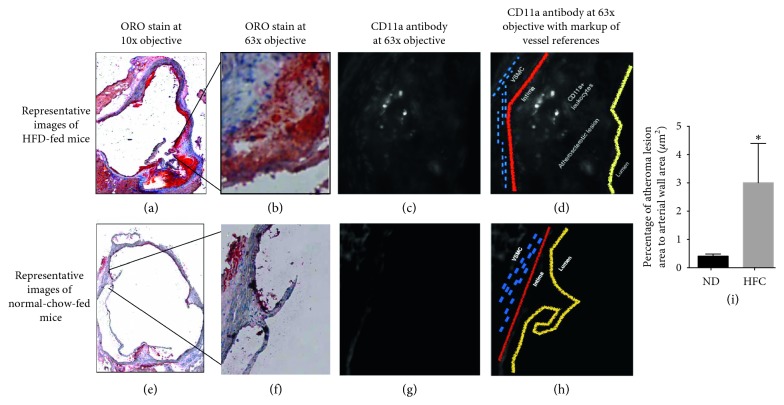
LFA-1 targeting in the aortic atherosclerotic plaque. Representative high degree atherosclerotic lesion is shown in HFD-fed mice from OCT subaortic leaflet atherosclerotic sections with a 10x (a) and 63x (b) objective of an Oil Red O stained section. The presence of LFA-1+ cells was correlated with epi fluorescent microscopy using FITC-conjugated rat anti-mouse CD11a antibody (c). A schematic illustration of the atherosclerotic plaque components in relationship to identified LFA-1+ cells (VSMC: vascular smooth muscle cells) is shown in (d). As a counterpart, representative 10x objective (e) and 63x objective (f) epi fluorescent microscopy of OCT frozen subaortic leaflet atherosclerotic sections show small lipid accumulation in the aortic sinus of normal-diet-fed mice. Included are representative images of FITC-conjugated rat anti-mouse CD11a antibody images (g) and a schematic illustration of the atherosclerotic plaque components in the immunohistochemistry image (h). apoE^−/−^ mice on a HFD exhibit increased percentage of atherosclerotic lesion to vessel wall area in comparison to normal-diet-fed mice (i). Two-tailed Student's *t*-test was used for statistical analysis (^*∗*^ *p* < 0.05) (*n* of 4 per group). ^*∗*^Immunohistochemistry was only assessed qualitatively but not quantitatively between groups.

Histologic analysis reflected accurate development of atherosclerotic lesions in all apoE^−/−^ mice by positive ORO tissue staining of OCT-embedded subaortic leaflets. The accumulation of inflammatory components in the subaortic leaflet region and lesion degree differed between experimental groups, especially shown in HFD-fed mice at 10x magnification (Figure 3) and at a 63x magnification (Figure 3). High-degree atherosclerotic lesions in HFD-fed apoE^−/−^ mice evidenced the presence of LFA-1+ leukocytes in the vascular atherosclerotic plaque (Figure 3). A scheme of the IHC images contrasted with the anatomical references (Figure 3) fused from representative images is shown in Figures 3 and 3. Oil Red O-stained cryosections from normal-diet-fed apoE^−/−^ mice showed low-degree atherosclerotic lesions (Figure 3) and also compared to HFD-fed apoE^−/−^ mice showed lower percentage of atherosclerotic lesion area in relationship to vessel wall area (*p* < 0.05) (Figure 3). These results confirm the enhanced disease development and inflammatory process in the vascular atherosclerotic plaque in apoE^−/−^ mice due to the HFD.

### 3.4. ^111^In-DANBIRT Molecular Imaging of LFA-1 Expression in Atherosclerosis Development

**Figure 4:**
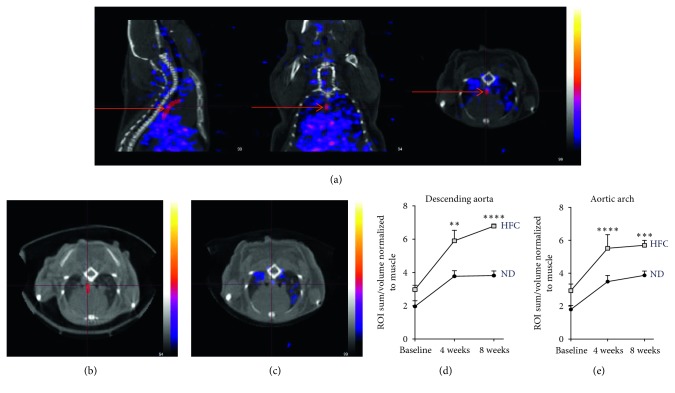
3-hour ^111^In-DANBIRT SPECT/CT imaging. Representative SPECT/CT image illustrates image analysis with the localization of the volume of the determined ROI in different imaging planes (axial, sagittal, and coronal), to correlate the SPECT date matched in this case to the descending aorta (red arrows depict the descending aorta) (a). Representative SPECT/CT 3D reconstruction shows low specific uptake and background ROIs in normal-diet-fed mice ((b); the image partially shows the determined ROI for the aortic arch in red color) compared to a HFD-fed mice (c) correlating with the increased atherosclerotic plaque development and identification of LFA-1+ cells inside of the plaque lesion by histologic analysis shown in Figure 3. Increased uptake of ^111^In-DANBIRT in the descending aorta (d) and aortic arch (e) of HFD-fed mice at baseline, 4 weeks, and 8 weeks. Repeated measures ANOVA with multiple comparisons was used for statistical analysis (^*∗∗∗*^ *p* < 0.001; ^*∗∗∗∗*^ *p* < 0.0001) (*n* of 4 per group).

**Figure 5:**
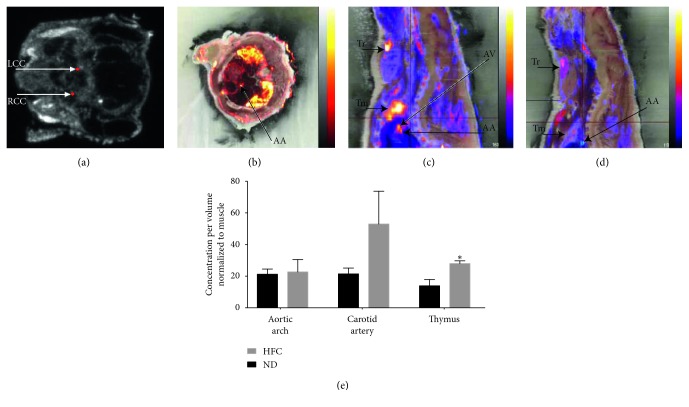
24-hour ^111^In-DANBIRT 3D autoradiography. (a) shows a representative image of the right and left common carotid arteries as they were clearly identified and isolated using determined ROI using fused autoradiography images to identify the left common carotid (LCC) and right common carotid (RCC) that were isolated and identified in red dots (white arrows point to the labeled ROI in each figure). (b-c) show representative images of ^111^In-DANBIRT uptake in HFD-fed mice in the aortic arch (AA) and aortic vessel outflow (AV) (black arrows point to the labeled ROI in each figure). (d) shows a representative image of ^111^In-DANBIRT uptake in normal-diet-fed mice in the aortic arch (AA) (black arrows point to the labeled ROI in each figure). Thymus (Tm) and thyroid (Tr) are shown as landmarks for anatomical references (black arrows point to the labeled ROI in each figure). (e) shows statistical analysis of the uptake of ^111^In-DANBIRT in the aortic arch and no difference between groups with increased ^111^In-DANBIRT uptake in HFD-fed mice even after 24 hours after injection in the carotid arteries (with no statistical significance) and the thymus (^*∗*^ *p* < 0.05). One-way ANOVA with multiple comparisons was used for statistical analysis (^*∗*^ *p* < 0.05) (*n* of 4 per group).

Representative SPECT/CT 3D reconstructions show longitudinal 3-hour ^111^In-DANBIRT SPECT/CT scans, which allowed us to identify atherosclerosis-prone ROI established in the aortic arch and the initial portion of the descending aorta tissues shown in the representative images displayed with the ROI volumes in red color that are being pointed by the red arrows (Figure 4(a)). Representative images are shown for the analysis performed on normal-diet-fed apoE^−/−^ mice, which evidenced the localized specific activity in the aortic arch delimited by the demarked cross pined red lines (Figure 4(b)). Representative images are also shown for the analysis performed on HFD-fed apoE^−/−^ mice, localizing the signal in the aortic arch identified in the cross pined red lines. Analysis of ROI uptake in different experimental groups identified an increased uptake in the descending aorta (Figure 4(d)) and aorta arch (Figure 4(e)) ROI evident at 4- and 8-week time points in HFD-fed compared to normal-diet-fed apoE^−/−^ mice by two-tailed Student' *t*-test statistical analysis (Figures 4(d) and 4(e)). 24-hour ^111^In-DANBIRT 3D autoradiography complemented the 3-hour SPECT/CT imaging ROI determination identifying uptake of radiolabeled DANBIRT in difficult areas. Specific uptake in the common carotid arteries (Figure 5(a)), aortic arch (Figure 5(b)), and aortic outflow tract (Figure 5(c)) was identified. As a positive control, an ROI for the thymus was delimited because of its rich leukocyte presence. The two-tailed Student's *t*-test showed a statistically significant increase of ^111^In-DANBIRT uptake in HFD-fed apoE^−/−^ mice compared to normal-diet-fed mice specifically in the thymus (Figure 5(d)), with increased uptake in the carotid arteries, although not statistically significant.

## 4. Discussion

Results of this study confirm (1) specificity of radiolabeled DANBIRT biodistribution in target tissues (Figure 1), illustrating a selective and competitive LFA-1 receptor binding in leukocytes (neutrophils) in an acute inflammatory exposure model (ozone) (Figure 2); (2) histopathologically observed development of aortic lesions (Figure 2) correlated with *in vivo* molecular imaging evidence of longitudinal increased uptake of ^111^In-DANBIRT in cardiovascular tissues in apoE^−/−^ mice (Figures 4 and 5); and (3) accurate characterization of atherosclerosis in a mouse model identifying early-stage development of vascular atherosclerotic plaque in cardiovascular tissues (Figure 3, Supplemental Figure 3).

*In vitro* stability studies with ^111^In-DANBIRT correlated to the findings by Poria et al. and its use by Mumaw et al. substantiate its potential as a molecular imaging probe. *In vitro* studies demonstrated high stability of ^68^Ga-DANBIRT throughout 4 hours of incubation with saline to mimic physiologic conditions for radiotracer delivery (Supplemental Figure 2), eliminating the possibility of having nonspecific binding to other blood proteins that could alter the radioligand incorporation yield. ^68^Gallium was used for DANBIRT radiolabeling for the *in vitro* experiments due to its short half-life and its availability in our laboratory. Rats were used rather than mice in order to minimize the need to pool blood due to the volume needed for the Histopaque cell isolation methods. Biodistribution experiments showed that liver had a significant amount of ^111^In-DANBIRT in both experimental groups. This finding is anticipated because of increased blood flow and immune cells in the liver, as well as radiotracer metabolism. As an important macroscopic finding, hepatic steatosis was evidenced in HFD-fed mice. This finding will guide future studies to understand such an effect. The targeted atherosclerosis-prone cardiovascular tissues and leukocytes had increased ^111^In-DANBIRT uptake in mice fed a HFD. As an observation, most tissues from apoE KO mice under a HFD had higher ^111^In-DANBIRT retention. It has been shown that apoE KO mice develop systemic inflammation, which is substantially potentiated by a high-fat diet [35, 36], which may explain why the apoE KO mice on a HFD show general higher ^111^In-DANBIRT uptake. Tissue uptake of radiolabeled DANBIRT is a measure of the quantity of inflammatory cells and the location and extent of the inflammatory process. The specificity of ^111^In-DANBIRT was evaluated *in vitro* under acute inflammatory conditions, as seen in early-stage atherosclerotic disease, demonstrating increasing ^111^In-DANBIRT uptake in neutrophils and monocytes. Rats were exposed to ozone, a proven animal model exhibiting local and systemic acute immune response in target cells [37]. The increased LFA-1 expression and corresponding high ^111^In-DANBIRT uptake in neutrophils following ozone exposure supports the concept of an acute systemic immune injury model.

The presence of ^111^In-DANBIRT in cardiovascular and immune tissues was evidenced by LFA-1 intraplaque expression from immunohistochemical assessment (Figure 3), while the systemic inflammatory response observed following exposure to inhaled ozone in the *in vitro* studies correlated to increased circulating leukocytes (Figures 2(a) and 2(b)). Macrophages are the most prevalent inflammatory cells within atherosclerotic plaques and have been hypothesized to be predictors of cardiovascular risk [38]. Therefore, resident macrophages and immature recruited neutrophils are suitable targets for noninvasive imaging techniques [39]. Inflammatory leukocytes have an important role of neutrophils in plaque vulnerability/instability and atherosclerosis progression via upregulation of vascular factors [18, 40, 41]. Leukocytes can be primed to increase the expression of LFA-1 and its affinity state.

The LFA-1/ICAM-1 complex is important in most vascular diseases because of its role in leukocyte recruitment and transmigration [42]. In order for LFA-1 to bind to ICAM1, LFA-1 must be activated to a high affinity state, a process which can be imaged via radiolabeled DANBIRT [33]. T-cell activation and migration to sites of inflammation is also guided predominantly by LFA-1/ICAM-1 interaction and Signal-2 [43], making the allosteric inhibition of LFA-1 an effective way to target intraplaque inflammation [33]. Molecular imaging of LFA-1 expressing leukocytes may help understand how modulation of this integrin impacts immune progression in a variety of diseases [44]. The results of this study show the ability of radiolabeled DANBIRT to specifically target LFA-1 to allow molecular imaging of inflammation in atherosclerosis in a mouse model.

*In vivo* SPECT/CT molecular imaging showed a longitudinally increased uptake in cardiovascular tissues in apoE^−/−^ mice fed a HFD compared to normal diet (Figures 4 and 5). Tissues surrounding the vessel lamina adventitia have been identified as sites of accumulation of inflammatory cells in apoE^−/−^ mice on normal diet [45]. This effect was not clearly identified in these studies due to limits of spatial resolution of the SPECT imaging system. 3D autoradiography was implemented in the study to allow both functional and advanced anatomical assessment of the presence of LFA-1 expression in cardiovascular tissues. The use of 3D autoradiography in molecular imaging provides increased spatial resolution compared to *in vivo* SPECT/CT. This improves the accuracy of colocalization of radiolabeled DANBIRT in target tissues and subsequently the identification of volumes of tissues determined for each ROI. Because of the specificity of DANBIRT for LFA-1's restricted expression in leukocytes and DANBIRT's small molecular weight, 3D autoradiography was performed at 24 hours to be able to evidence the clearance of the tracer mostly from the bloodstream and major vessels after 3-hour SPECT/CT imaging. Correlation of SPECT/CT imaging 24 hours with autoradiography showed increased uptake in the resident-leukocyte-rich thymus, confirming functional and morphological distribution of 111In-DANBIRT. 3D autoradiography was critical, allowing radioligand localization in areas difficult for identification using plain CT or SPECT/CT because of the interference in the signal coming from small vessels, and the limits of detector sensitivity and specificity [46].

The ability of DANBIRT to assess and quantify *in vivo* inflammation presents an opportunity to functionally characterize developing atheroma and atherosclerosis. Current molecular imaging techniques such as FDG-PET/CT localize general metabolic activity but lack specificity for molecular events associated with inflammatory cell activation [5, 47].

## 5. Conclusions

Functional and morphological assessment of atherosclerotic plaque using ^111^In-DANBIRT relies on LFA-1's restricted expression in leukocytes and DANBIRT's low molecular weight and specificity. Longitudinal *in vivo* imaging of the inflammatory process in atherosclerotic plaque is very promising and will allow accurate and timely risk assessment of cardiovascular-related complications [48]. ^111^In-DANBIRT is a promising molecular imaging probe targeting LFA-1 in the inflammatory process of vascular atherosclerotic lesions. Future investigations will be guided towards utilizing radiolabeled DANBIRT in other small animal models of vascular disease to further study its capacity and limitations. Future studies will be aimed to advance the potential of DANBIRT as a preclinical molecular imaging probe.

## Data Availability

The source data are stored at the University of New Mexico and could be made available from the corresponding author upon request.

## Supplementary Materials

Supplementary Materials Supplemental Figure 1: chemical structure of DANBIRT. DANBIRT is a chemically restructured radioligand capable of binding to LFA-1 expressed by leukocytes (M.W. 885.6 G). DOTA: 1,4,7,10-tetraazacyclododecane-1,4,7,10-tetraacetic acid butylaminoNorBIRT: (R)-1-(4-aminobutyl)-5-(4-bromobenzyl)-3-(3,5-dichlorophenyl)-5-methylimidazolidine-2,4-dione BIRT 377: (R)-5-(4-bromobenzyl)-3-(3, 5-dichlorophenyl)-1,5-dimethylimidazolidine-2,4-dione DANBIRT: DOTA-Alkylamino-NorBIRT: [Radiometal]-DOTA-1-(4-aminobutyl)-5-(4-bromobenzyl)-3-(3,5-dichlorophenyl)-5-methylimidazolidine-2,4-dione. Supplemental Figure 2: radiolabeled DANBIRT *in vitro* stability. (A) Radiolabeled DANBIRT samples ran in triplicate were incubated in FBS and 0.9% saline solution, showing >95% mean radiochemical purity and incorporation yield at baseline, 5 minutes, 30 minutes, 60 minutes, 120 minutes, and 240 minutes after incubation. (B) Representative ITLC image result showing incorporation yield; peak represents ROI incorporation yield percentage >99%. Supplemental Figure 3: characterization of vascular atherosclerotic disease model. (a) Percentage of weight change from baseline compared weekly during 8 weeks of dietary assessment, showing an increase in body weight in HFD-fed mice clearly evident after 4-week time point. (b) Serum total cholesterol and (c) total triglyceride levels showed a difference between dietary groups. (d) VLDL-cholesterol was significantly higher in HFD-fed mice. (e) HDL-cholesterol did not show a difference between both groups. Concentration is represented in milligrams per deciliter (mg/dL) and in nanometers (nm) for particle size. Two-sided *t*-test and one-way ANOVA with multiple comparisons were used for statistical analysis (*n* of 4 per group). Asterisks represent statistical significance (^*∗*^ *p* < 0.05; ^*∗∗*^ *p* < 0.01; ^*∗∗∗∗*^ *p* < 0.0001). Supplemental Table 1: UPLC method gradient with flow rate. Supplemental Table 2: [111In] In-DANBIRT animal study design. apoE^−/−^ mice were exposed for 8 weeks to either normal chow or high-fat chow for experiment samples. Sprague-Dawley rats were exposed to ozone or filtered air for 4 hours. Supplemental Table 3: SPECT/CT imaging parameters.
