# Supplementary material for: 111In-DANBIRT In Vivo Molecular Imaging of Inflammatory Cells in Atherosclerosis
Source: Contrast Media Mol Imaging. 2018 Nov 13;2018:6508724. doi: 10.1155/2018/6508724 (PMC6257909; doi:10.1155/2018/6508724)
Supplement: Supplementary Materials — Supplemental Figure 1: chemical structure of DANBIRT. DANBIRT is a chemically restructured radioligand capable of binding to LFA-1 expressed by leukocytes (M.W. 885.6 G). DOTA: 1,4,7,10-tetraazacyclododecane-1,4,7,10-tetraacetic acid butylaminoNorBIRT: (R)-1-(4-aminobutyl)-5-(4-bromobenzyl)-3-(3,5-dichlorophenyl)-5-methylimidazolidine-2,4-dione BIRT 377: (R)-5-(4-bromobenzyl)-3-(3, 5-dichlorophenyl)-1,5-dimethylimidazolidine-2,4-dione DANBIRT: DOTA-Alkylamino-NorBIRT: [Radiometal]-DOTA-1-(4-aminobutyl)-5-(4-bromobenzyl)-3-(3,5-dichlorophenyl)-5-methylimidazolidine-2,4-dione. Supplemental Figure 2: radiolabeled DANBIRT in vitro stability. (A) Radiolabeled DANBIRT samples ran in triplicate were incubated in FBS and 0.9% saline solution, showing >95% mean radiochemical purity and incorporation yield at baseline, 5 minutes, 30 minutes, 60 minutes, 120 minutes, and 240 minutes after incubation. (B) Representative ITLC image result showing incorporation yield; peak represents ROI incorporation yield percentage >99%. Supplemental Figure 3: characterization of vascular atherosclerotic disease model. (a) Percentage of weight change from baseline compared weekly during 8 weeks of dietary assessment, showing an increase in body weight in HFD-fed mice clearly evident after 4-week time point. (b) Serum total cholesterol and (c) total triglyceride levels showed a difference between dietary groups. (d) VLDL-cholesterol was significantly higher in HFD-fed mice. (e) HDL-cholesterol did not show a difference between both groups. Concentration is represented in milligrams per deciliter (mg/dL) and in nanometers (nm) for particle size. Two-sided t-test and one-way ANOVA with multiple comparisons were used for statistical analysis (n of 4 per group). Asterisks represent statistical significance (∗ p < 0.05; ∗∗ p < 0.01; ∗∗∗∗ p < 0.0001). Supplemental Table 1: UPLC method gradient with flow rate. Supplemental Table 2: [111In] In-DANBIRT animal study design. apoE−/− mice were exposed for [file 6508724.f1.pdf]

Supplemental

Title: [111In] In-DANBIRT *In Vivo* Molecular Imaging of Early-Stage Atherosclerosis: Identification of LFA-1+ leukocytes in the Intraplaque Inflammatory Process

Short running title: Imaging Inflammation in Atherosclerosis

Roberto Mota[1,2], Matthew J. Campen[3], Matthew E. Cuellar[4] , William S. Garver[5], Jacob Hesterman[6], Mohammed Qutaish[6], Tamara Daniels[1], Monique Nysus[1], Carston R. Wagner[4], Jeffrey P. Norenberg[1,7]

1. Radiopharmaceutical Sciences, University of New Mexico (UNM), Albuquerque, NM
2. Department of Surgery, Division of Vascular Surgery, University of North Carolina at Chapel Hill, Chapel Hill, NC (current).
3. Pharmaceutical Sciences, UNM, Albuquerque, NM
4. Medicinal Chemistry, College of Pharmacy, University of Minnesota, Minneapolis, MN
5. Department of Biochemistry & Molecular Biology, School of Medicine, UNM, Albuquerque, NM
6. InviCRO, Boston, MA
7. Department of Anesthesiology and Critical Care Medicine, School of Medicine, UNM, Albuquerque, NM Running Title: Imaging Inflammation in Atherosclerosis

Corresponding Author:

Jeffrey P. Norenberg, PharmD, PhD, FASHP, FAPhA, FACNM

2502 Marble Ave, NE; MSC 09 5360

Albuquerque, NM 87131-0001

email: jnorenberg@salud.unm.edu Telephone: 505.272.8101

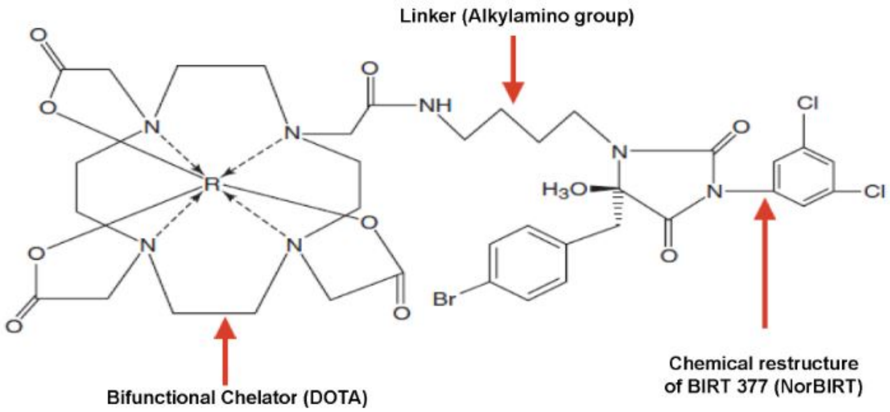

Supplemental Fig 1. Chemical structure of DANBIRT. DANBIRT is a chemically restructured radioligand capable of binding to LFA-1 expressed by leukocytes (M.wt 885.6G). DOTA: 1,4,7,10-tetraazacyclododecane- 1,4,7,10-tetraacetic acid butylaminoNorBIRT: (R)-1-(4-aminobutyl)-5-(4-bromobenzyl)-3-(3,5-dichlorophenyl)-5- methylimidazolidine-2,4-dione BIRT 377: (R)-5-(4-bromobenzyl)-3-(3, 5-dichlorophenyl)- 1,5-dimethylimidazolidine-2,4-dione DANBIRT: DOTA-Alkylamino-NorBIRT: [Radiometal]-DOTA-1-(4-aminobutyl)-5-(4-bromobenzyl)-3-(3,5-dichlorophenyl)-5- methylimidazolidine-2,4-dione

A

	HPLC/ITLC Baseline		HPLC/ITLC 5 min		HPLC/ITLC 30 min		HPLC/ITLC 60 min		HPLC/ITLC 120 min		HPLC/ITLC 240 min	
FBS pH 7-8	100	96.4	99.98	97.6	100	97.2	99.99	97.2	99.96	97.4	99.99	97.4
Sample 3			100		100		100		99.9		100	
Sample 2			100		100		99.98		100		99.99	
Sample 1			99.96		100		100		99.98		99.98	
0.9% NaCl pH ~5.5	99.99	98.5	99.95	96	99.96	96	99.94	97.9	99.94	95.63	99.8	97.99
Sample 3			NR		99.94		100		99.93		99.99	
Sample 2			100		99.97		99.96		99.91		100	
Sample 1			99.9		99.98		99.86		99.98		99.42	

B

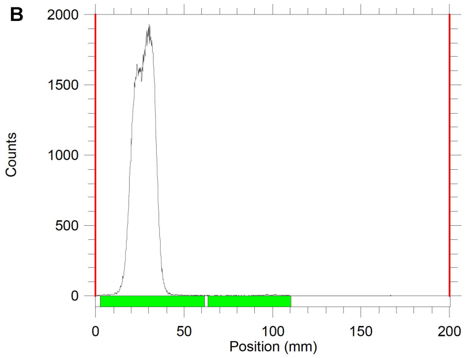

Supplemental Fig 2. Radiolabeled DANBIRT in vitro stability. (A) Radiolabeled DANBIRT samples ran in triplicate were incubated in FBS and 0.9% Saline solution, showing >95% mean radiochemical purity and incorporation yield at baseline, 5 minutes, 30 minutes, 60 minutes, 120 minutes and 240 minutes post incubation. (B) Representative ITLC image result showing incorporation yield, peak represents ROI incorporation yield percentage >99%.

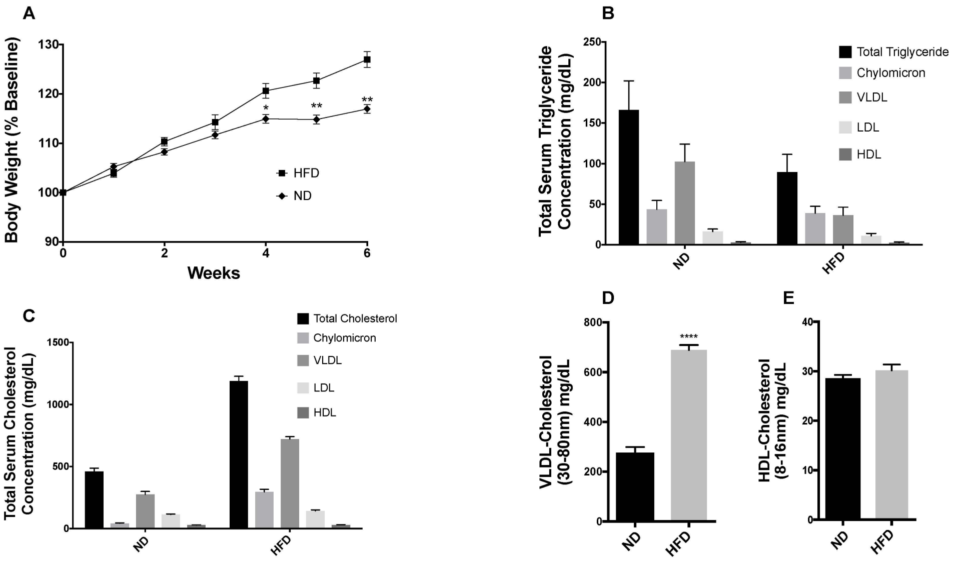

Supplemental Fig 3. Characterization of vascular atherosclerotic disease model. (A) Percentage of weight change from baseline compared weekly during 8 weeks of dietary assessment, showing an increase in body weight in HFD-fed mice clearly evident after 4 week time point. (B) Serum total cholesterol and (C) total triglyceride levels showed a difference between dietary groups. (D) VLDL-Cholesterol was significantly higher in HFD-fed mice, (E) HDL-Cholesterol did not show a difference between both groups. Concentration is represented in milligrams per deciliter (mg/dL) and in nanometers (nm) for particle size. Two-sided t-test and One-Way ANOVA with multiple comparisons were used for statistical analysis (n of 4 per group). Asterisks represent statistical significance (*p<0.05, **p<0.01, ****p<0.0001).

	Time (min)	% Solvent B	Flow (ml/min)
1	0.00	50.0	0.800
2	0.15	50.0	0.800
3	3.00	60.0	0.800
4	5.00	80.0	0.800
5	7.00	100.0	0.800
6	7.50	100.0	0.800
7	8.00	0.0	0.800
8	8.50	0.0	0.800
9	8.60	50.0	0.800
10	9.00	50.0	0.800

Supplemental Table 1. UPLC method gradient with flow rate.

ApoE$^{-/-}$ mice (C57BL/6 background): n of 24			
	Normal Diet	High Fat Diet (HFC)	Total
SPECT/CT, Bio distribution	4	4	8
Serum lipids	4	4	8
3D Autoradiography	4	4	8
Total	12	12	24

Sprague Dawley male rats: n of 6		
	Filtered Air	Ozone (1ppm)
Ozone vs Filtered Air 4-hr Exposure	3	3

Supplemental Table 2. [111In] In-DANBIRT animal study design. apoE-/- mice were exposed for 8 weeks to either normal chow or high fat chow for experiment samples. Spargue Dawley rats were exposed to ozone or filtered air for 4 hours.

Topogram/CT				SPECT	
Scan Range (mm)	~30	Projections	360	Pinhole Size (mm)	1.4
				Projections/Rotation	360
Tube voltage (kVp)	65	Pitch	1.5	Time/Projection (s)	~75
Exposure time (ms)	500	Acquisition time (min:sec)	3:00	Acquisition time (min:sec)	~60:00
Tube current (µA)		123		Energy window (keV)	158-183, 226-263

Supplemental Table 3. SPECT/CT imaging parameters
